# Reply to ‘Pitfalls of using confocal-microscopy based automated quantification of synaptic complexes in honeybee mushroom bodies (*responseto Peng and Yang 2016*)’

**DOI:** 10.1038/s41598-017-11858-x

**Published:** 2017-09-07

**Authors:** Yi-Chan Peng, En-Cheng Yang

**Affiliations:** 1 Department of Entomology, National Taiwan University, Taipei, Taiwan; 2 Graduate Institute of Brain and Mind Sciences, National Taiwan University, Taipei, Taiwan

## Abstract

In a comment on our Article “Sublethal Dosage of Imidacloprid Reduces the Microglomerular Density of Honey Bee Mushroom Bodies”, Rössler *et al*. assert that our reported counts are overall lower than previously reported due to the use of automated quantification. We address these issues in this reply.

## Introduction

The earlier study reported by Groh *et al*.^1^ did not quantify the uneven distribution of the microglomeruli in the calyces, even though this appeared in their micrographs. We noticed that 4 selected areas within the so-called dense region of Fig. 1B clearly have more dense labelling when compared to the unselected central area^1^. This effect is also apparent in Fig. 1B in the Rössler *et al*. paper. Thus it seems clear that just by choosing a particular region of interest (Fig. 1B for confocal microscopy and Fig. 3A for EM in Groh *et al*.^1^) would cause bias. It was for this very reason therefore, that the entirety of the calyces was counted in our study. It is thus reasonable that our results showed that the densities are overall lower compared with previous reports due to the particular regions selected and methods used in those studies. In summary, earlier methods that assumed uniform density are difficult to compare with our method that comprehensively measured all regions, and thereby quantified the regional differences for the first time.

In our study, each calyce underwent a thorough scanning of the entire structure, and were later processed by software to count MGs^2^. The image was three-dimensional and was thus difficult to present how the MGs were distributed. Therefore, we chose as an example only one layer of the captured image to indicate the phenomenon. This one layer does not represent the whole picture of the distribution of microglomeruli in the calyces. The Fig. 1 in Rössler *et al*.’s paper used our example figure and put a yellow box on it intending to show the density of microglumerli in a volume of 10 μm × 10 μm × 10 μm. We do not think this is a proper method for comparison given that much less than the whole data set is used, the authors, again, ignore the uneven distribution of the microglomeruli in the calyces.

We were aware that the densities reported in our study are lower than previous research. But this contrast may be caused by the different counting methods, sampling methods and different populations etc. The foragers in our study were the ones carrying pollen on their corbiculate legs we collected outside the hive. Those foragers are elder than 20 days but younger than 30. It is possible that the young forgers of our study had a lower number of microglomeruli when compared with 35-day-old foragers in Groh *et al*.^1^. The purpose of our study was to compare the MG number and density before and after the imidacloprid treatment during the larval stage. Therefore, we must build new baselines for every region of the calyx so that we could compare before and after applying the same method and analyze the data statistically. The honey bees fed with sublethal dosages certainly show lower density of microglomeruli in the calyces than normal ones. The results showed a profound impact on their nervous system.
